# Novel hemostatic biomolecules based on elastin-like polypeptides and the self-assembling peptide RADA-16

**DOI:** 10.1186/s12896-018-0422-5

**Published:** 2018-03-07

**Authors:** Shasha Yang, Sili Wei, Yun Mao, Hanxue Zheng, Juantao Feng, Jihong Cui, Xin Xie, Fulin Chen, Honmgmin Li

**Affiliations:** 1 Lab of Tissue Engineering, Provincial Key Laboratory of Biotechnology of Shaanxi, College of Life Sciences, Northwest University, Taibai North Rd 229, Xi’an, Shaanxi Province 710069 China; 2 Key Laboratory of Resource Biology and Biotechnology in Western China, Ministry of China, Taibai North Rd 229, Xi’an, Shaanxi Province 710069 China

**Keywords:** Hemostatic material, ITC, His-tag, Affinity chromatography, Purification, Collagen

## Abstract

**Background:**

Safe and effective hemostatic materials are important for reducing mortality resulting from excessive hemorrhage. In this work, new biomaterials with hemostatic effects were created by fusing the gene coding for RADA-16, a self-assembling peptide with the sequence RADARADARADARADA, to the 3′-end of the open reading frame (ORF) encoding elastin-like polypeptides through gene recombination.

**Results:**

The fusion proteins, termed 36R, 60R and 96R, were solubly over-expressed in *Escherichia coli* BL21 (DE3) based on genetic manipulation of the high-efficiency prokaryotic expression vector pET28a (+) and bacterial transformation. Western Blot analysis showed that the over-expressed proteins were the target fusion proteins. The target proteins 36R with 94.72% purity, 60R with 96.91% purity and 96R with 96.37% purity were prepared using an inverse phase transition cycle at 65 °C followed by His-tag affinity chromatography. The proliferation results of the mouse fibroblast cell line L929 and hippocampus neuron cell line HT22 indicated that the fusion proteins did not cause obvious cell toxicity. The lyophilized spongy film of the purified 36R, 60R and 96R could stop the hemorrhage of a 2 × 2 mm bleeding wound in the mouse liver after 27.21 ± 1.92 s, 18.65 ± 1.97 s and 15.85 ± 1.21 s, respectively. The hemostasis time was 21.23 ± 1.84 s for rat-tail collagen and 14.44 ± 1.33 s for RADA-16 lyophilized on gauze. The hemostatic time of three treated groups were all significantly superior to that of the negative control without any hemostasis treatment, which spontaneously stopped bleeding after 37.64 ± 1.34 s. Statistical analysis showed that the spongy film with purified 96R exhibited an exciting hemostatic effect that was superior to rat-tail collagen and close to that of RADA-16 lyophilized on gauze.

**Conclusions:**

These results revealed that the fusion proteins achieved by gene recombination technology could serve as a promising hemostatic material.

**Electronic supplementary material:**

The online version of this article (10.1186/s12896-018-0422-5) contains supplementary material, which is available to authorized users.

## Background

Fast, effective hemostasis is critical for reducing mortality due to massive hemorrhage in accidental injury or surgery. In the event of hemorrhage, natural hemostasis is slow and complicated, including many processes such as vasal contraction, platelet aggregation and progressive activation of coagulation factors. For some cases, it is impossible to wait for the natural hemostatic process to occur. Therefore, additional methods for stable coagulum must be applied [1, 2]. In addition to the strong effects of mechanical compression, thermal cauterants or preventative chemical regents such as aprotinin [3] and minirin [4], hemostatic materials play important roles in blocking bleeding. QuickClot is an effective hemostatic material produced by the American manufacturer Z-medica whose main component is zeolite. QuickClot can quickly prevent blood loss and sharply decrease the mortality resulting from excessive hemorrhage [5], but its safety depends on prudent use, as zeolite can give rise to local heat shock (or local overheating). Similar to zeolite, polysaccharide hemostatic materials including chitosan, starch and cellulose can rapidly adhere to wounds and absorb moisture, accelerate the formation of blood clots and stop bleeding. Another prominent protein-based hemostatic material is fibrin glue, which contains thrombin and can effectively accelerate the activation of coagulation factors to stop bleeding [6].

In recent years, nanometer materials with self-assembling character have evoked keen interest in biomaterials research. RADA-16, a synthesized peptide with the sequence AcN-ArgAlaAspAlaArgAlaAspAlaArgAlaAspAlaArgAlaAspAla -CONH2 [7], possesses self-assembling properties. The amino acid residues Arg and Asp appear alternately in the sequence of RADA-16, imparting RADA-16 with amphipathic properties that make it highly prone to organize into stable β-sheet structures and form nanofibers with 10–15 nm diameters in a solution with 0.1–1% (*w*/*v*) concentration. RADA-16 nanofibers can elongate to 200–400 nm in solution with pH 4.0–9.5, and the density of mesh increases with increasing concentration [8, 9]. When 1% (w/v) RADA-16 aqueous solution is added to a bleeding wound in brain, spinal cord, femoral artery, liver or skin, RADA-16 peptides can quickly self-assemble to form a layer of nanometer fibers, which act as barrier to effectively block hemorrhage in less than 15 s [10, 11]. More attractively, the hemostasis does not depend on compression, thermal cauterant or preventative chemical regents. In addition, the degradation product of RADA-16 is amino acids which can be used by the organism to repair wound tissue [12]. The biocompatibility and safety of RADA-16 is accepted as a biomaterial [13]. A promising hemostatic commodity, the PuraStat® pre-filled syringe with RADA-16 as the effective component, has been developed and is sold by 3-D Matrix Medical Technology [14]. Chemical synthesized RADA-16 is safe, non-biogenic and without the risk of transmissible spongiform encephalopathy transmission. However, some intermediate impurity is difficult to remove for solid-phase synthesis, and liquid-phase synthesis needs lengthy recrystallization and multi-step column chromatography. As a result, the cost is relative high, about 2800 RMB for 50 mg of synthesized RADA-16 in China. Genetically engineered preparation is a promising alternative method to obtain pharmaceutical proteins with low-cost. But, this way is not adaptable for the peptides with low molecular weight (MW) as the poor stability of target peptides under the proteases attack in host cells make it hardly to detect. Fusion protein strategy provide a way to improve the stability of small MW peptide [15].

Elastin-like polypeptides (ELPs) are composed of repeats of VPGXG or VAPGXG (X can be any amino acid other than Pro) that are genetically conjugated in series. The former appears in cow elastin and the latter appears in human elastin with high frequency. Elastin-like polypeptides have promising properties which exhibit many possible applications in protein purification, bio-sensing, nanoassembly and therapeutic medicine [16]. Elastin-like polypeptides have been extensively investigated in cell culture, tissue repair or drug delivery [17–19]. Interestingly, the properties of ELPs can be retained after fusing with other peptides. Agnes reported that the bioactivity of the genetically fused protein SDF1α-ELP in vivo was significantly superior to that of free SDF1 [20]. TNF-VHH-ELP fusion proteins effectively prevent death caused by septic shock, and the in vivo persistence of TNF-VHH-ELP was 24-fold longer than that of TNF-VHH [21]. In addition, several bioactive ELP fusion proteins have been developed for different application purposes [22, 23]. These reports indicate that more fusion proteins with specific function can created through gene recombination technology with ELPs as substrate materials. Here, we reported novel hemostasis molecules, and human elastin-like polypeptide fusion RADA-16 (hELPs-RADA-16) was genetically prepared using transgenic *Escherichia coli* (*E. coli*).

## Methods

### Construction of expression vector

Restriction endonucleases and other enzymes used for gene manipulation were purchased from TaKaRa Biolabs unless otherwise mentioned. The cloning vectors pMD19-T-hELP36, pMD19-T-hELP60 and pMD19-T-hELP96, were constructed and retained by our lab, and these three vector contained the gene coding for hELP36 with the formulation of MGRS ((VAPGVG)_12_S)_3_, hELP60 with the formulation of MGRS ((VAPGVG)_12_S)_5_ and hELP96 with the formulation of MGRS ((VAPGVG)_12_S)_8,_ respectively. Each of these hELP-encoding sequences has the recognition sites of *Nco*I followed directly by *Bgl*II at the 5′ end and *Bam*HI followed directly by *Xho*I at the 3′ end. The gene coding for RADA-16 with 5′-end-*Bam*HI, followed by the coding sequence of GGS, and 3′-end-*Xho*I was synthesized. After *Bam*HI and *Xho*I double digestion, fragments of the synthesized gene of RADA-16 were respectively recombined with pMD19-T-hELP36, pMD19-T- hELP60 and pMD19-T-hELP96 using T_4_ DNA ligase. For every ligation treatment, 4 transformed colonies were randomly selected, cultured in liquid lysogeny broth (LB) media and plasmids were extracted, from which three recombinant cloning vectors pMD19-T-hELP36- RADA-16, pMD19-T-hELP60-RADA-16 and pMD19-T- hELP96-RADA-16 were selected and identified by restriction endonuclease digestion and sequencing. The coding sequences of hELP36-RADA-16, hELP60-RADA-16 and hELP60-RADA -16 were excised from the recombinant cloning vectors with *Nco* I and *Xho*I double digestion and subcloned into the prokaryotic expression vector pET28a (+). Three expression vectors pET28a (+)-hELP36-RADA-16, pET28a (+)-hELP60-RADA-16 and pET28a (+)-hELP96-RADA-16 were obtained after restriction endonuclease digestion, each of them was selected from 4 transformed colonies picked randomly.

### Protein expression

The plasmids of pET28a (+)-hELP36-RADA-16, pET28a (+)-hELP60-RADA-16 and pET28a (+)-hELP96-RADA-16 were used to transform the competent cells of *E. coli* BL21 (DE3) and spread on LB solid media containing 100 μg/mL kanamycin. After 12 h of culture, a single colony was selected and cultivated in LB liquid media containing 100 μg/mL kanamycin. The working solution of 100 μg/mL isopropylthio-β-D-galactoside (IPTG) was added to the culture mixture to induce target gene expression for 6 h, and the target polypeptides were termed 36R, 60R and 96R, respectively. The induced cells were collected and lysed by ultrasonication at 4 °C. The expression of target protein was visualized by SDS-PAGE. The lysis of non-induced *E. coli* BL21 (DE3) and *E. coli* BL21 (DE3) transformed by pET28a (+) were employed as negative controls.

### Solubility analysis and purification

The IPTG-induced cells of *E. coli* BL21 (DE3) were collected, resuspended in double-distilled water, and lysed completely with ultrasonication at 4 °C.Cell lysis were centrifuged at 12,000 rpm for 10 min. The supernatant was used to analyze the solubility of target proteins by SDS-PAGE, and the precipitate was resuspended in same volume and used to investigate whether the target proteins were expressed and accumulated in the form of inclusion bodies in the host cells. Subsequently, the portion containing target proteins was collected for purification by inverse phase transition cycles (ITC) methods.

### Western blotting analysis

Cell lysis containing recombinant 36R, 60R or 96R was separated by SDS-PAGE and transferred onto a polyvinylidene fluoride membrane (PVDF). The PVDF membrane was activated with 5–10s treatment of methyl alcohol and incubated with a solution of mouse monoclonal antibody against His-tag (abcam, America). The PVDF membrane was washed three times using phosphate buffer saline containing 0.05% Tween20 for fifteen minutes each and incubated with goat anti-mouse IgG-conjugated horseradish peroxidase antibody (KangWei, China). Peroxidase activity was detected using an electrochemiluminescence solution (Millipore) and captured in a gel imaging system (GE, image Qoant 350*).

### Protein purification and membrane preparation

ITC was employed to purify the target proteins 36R, 60R and 96R [24]. The practical transition temperature (Tt) of the target protein was obtained by a preliminary experiment (Cell lysis was treated at different temperatures; the temperature at which the target protein in cell lysis could quickly aggregate to form precipitated phase was selected for phase transition). In the first round of ITC, the target proteins were treated at 65 °C to trigger the phase transition. After 10 min centrifugation at 12,000 rpm, the precipitate containing target proteins was resuspended in Tris-HCl buffer (20 mM Tris-HCl, pH 8.0, 300 mM NaCl, 0.1 mM ethylene diamine tetraacetic acid, 10 mM imidazole and 1 mM phenylmethane- sulfonyl fluoride) for affinity purification according to the His-tag purification protocol [25]. Dialysis desalting was conducted before vacuum freeze-drying. The purified protein solution was transferred to 6-cm poly-tetrafluoroethylene dishes, quick-frozen at − 80 °C for 12 h and lyophilized in a CHRIST freeze-dryer (Beijing Bo Yi Kang Experimental Instrument Company, China) under vacuum for 48 h. The membranes of lyophilized proteins were cut into 0.8 × 0.8 cm pieces for the hemostasis experiment.

### Cell culture and growth assay

At first, it was determined that the IC_50_ of 36R, 60R, 96R was 2.5 μg/mL, 2.0 μg/mL and 2.0 μg/mL respectively based on three repetitive pre-experiments. The mouse fibroblast cell line L929 and hippocampus neuron cell line HT22 were cultured in Dulbecco’s Modified Eagle’s Medium (DMEM) containing 10% fetal bovine serum and antibiotics (400 U/mL penicillin, 400 μg/mL streptomycin) at 37 °C, 5% CO_2_ and 95% humidity. After determination using a BCA (bicinchoninic acid) protein assay kit, the concentration of recombinant proteins 36R, 60R and 96R was adjusted to 1.5 μg/mL using ultrapure water and sterilized with a 0.22-um filter membrane. Filtration sterilized recombinant proteins 36R, 60R, 96R, rat-tail collagen and RADA-16 (1.5 μg/mL) were coated into a 96-well plates. L929 and HT22 cells were planted into the coated 96 cell plate at a density of 3000 cells/well. The cell proliferation was measured using the Cell Counting Kit (CCK-8) (DOJINDO, Japan) after 12 h, 24 h, 36 h, 48 h, 60 h and 72 h culture according to the manufacture’s instruction. The morphological observation was conducted under microscopy simultaneously [26].

### Mouse liver hemostasis

All the mice were purchased from the animal center laboratory at the Fourth Military Medical University, China. The Northwest University Animal Care and Use Committee approved all procedures involving animals. Mice housing and breeding were conducted according to the recommendations of “The use of non-human primates in research”. Forty-two mice were divided into six groups, with 7 mice in each group. Mice were anesthetized by intraperitoneal injection of 10% urethane and their abdominal cavities were opened to exposure and place the liver on sterile gauze. At approximately 2 mm depth, a 2 mm diameter wound was quickly created using a processed syringe needle. After spontaneous bleeding for 3 s, blood was suctioned from the wound surface, the protein membrane was applied to the bleeding site, and the hemostatic time was recorded immediately with second chronograph. Rat-tail collagen sponge and 1% RADA-16 lyophilized on gauze acted as positive controls. Negative controls received no processing.

### Statistical analysis

All experiments were repeated at least 3 times. For cell culture and growth assay, and mouse liver hemostasis experiments, statistical analysis was conducted using SPSS 22 software, the difference between treatment groups was analyzed based on one-way analysis of variance (ANOVA).

## Results

### Construction of clone vectors

**Fig. 1:**
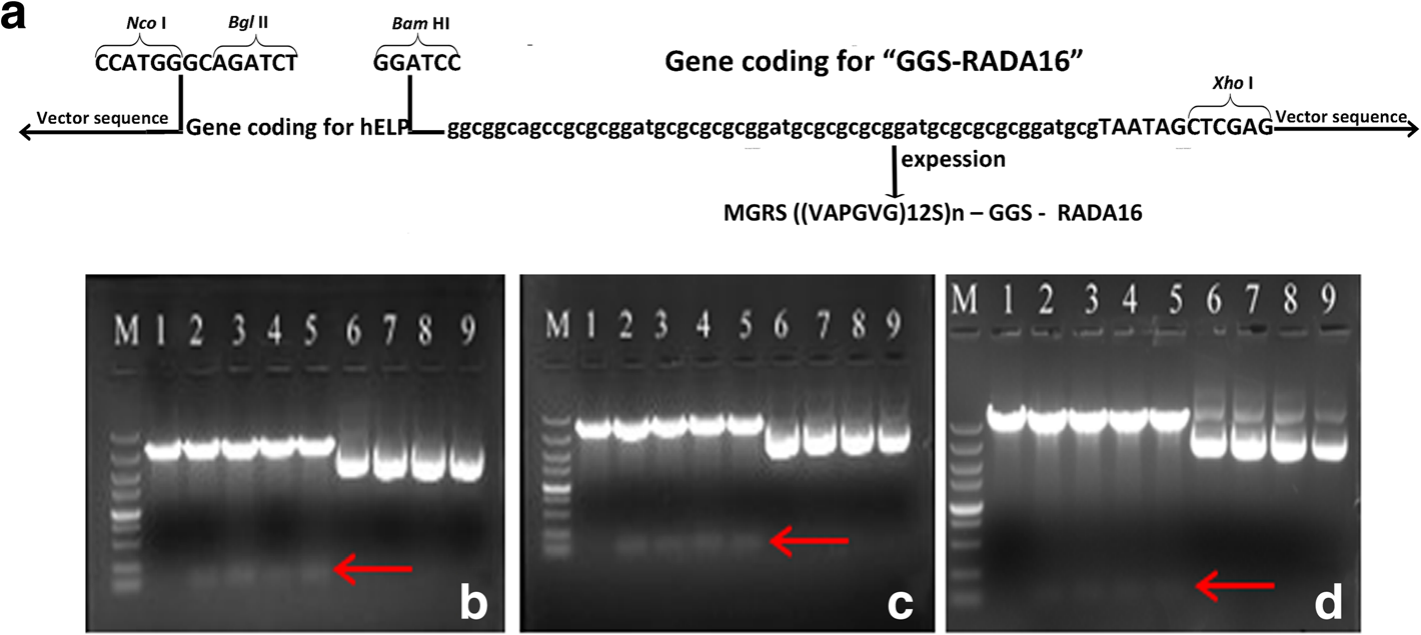
Construction design and restriction endonuclease analysis of clone vectors of hELPs-RADA16. M: DL5000 (**a**): The sequence information of designed fusion protein; **b**1: pMD19-T-hELP36 digested by *Bam*HI & *Xho*I; **b**2–5: Restriction endonuclease digestion product of pMD19-T-hELP36R prepared from four transformed colonies; **b**6–9: pMD19-T-hELP36R plasmids DNA corresponding to **b**2–5; **c**1: pMD19-T-hELP60R digested by *Bam*HI & *Xho*I; **c**2–5: Restriction endonuclease digestion product of pMD19-T-hELP60R prepared from four transformed colonies; **c**6–9: pMD19-T-hELP60R plasmids DNA corresponding to **c**2–5; **d**1: pMD19-T-hELP96R digested by *Bam*HI & *Xho*I; **d**2–5: Restriction endonuclease digestion product of pMD19-T-hELP96R prepared from four transformed colonies; **d**6–9: pMD19-T-hELP96R plasmids DNA corresponding to **d**2–5

To achieve some novel hemostatic biomaterials, several fusion proteins were designed based on self-assembling peptide RADA-16 and hELPs with ITC property (Fig. 1a). To obtain the biomolecules, RADA-16 coding sequences composed of optimized codons in accordance with the codon bias of *E. coli* were fused to the 3′-end of the open reading frame (ORF) of hELP36, hELP60 and hELP96 using the restriction endonuclease recognition sites of *Bam*HI at 5′-end and *Xho*I at the 3′-end of RADA-16-encoding sequence, as well as the *Bam*HI & *Xho*I sites at the 3′-end of the gene coding for hELPs. The double-digestion result of pMD19-T-hELP36- RADA-16, pMD19-T-hELP60-RADA-16 or pMD19-T-hELP96R-RADA-16 showed an about 100 bp product marked with red arrows, suggesting that the three clone vectors contain the synthesized ORF coding for 36R, 60R and 96R, respectively (Fig. 1b–d). The sequencing identification further certifies the three clone vectors were successfully obtained, in which the ORFs of fusion proteins hELPs- RADA-16 were correct (sequencing results were provided as Additional files 1, 2 and 3).

### Construction of expression vector

**Fig. 2:**
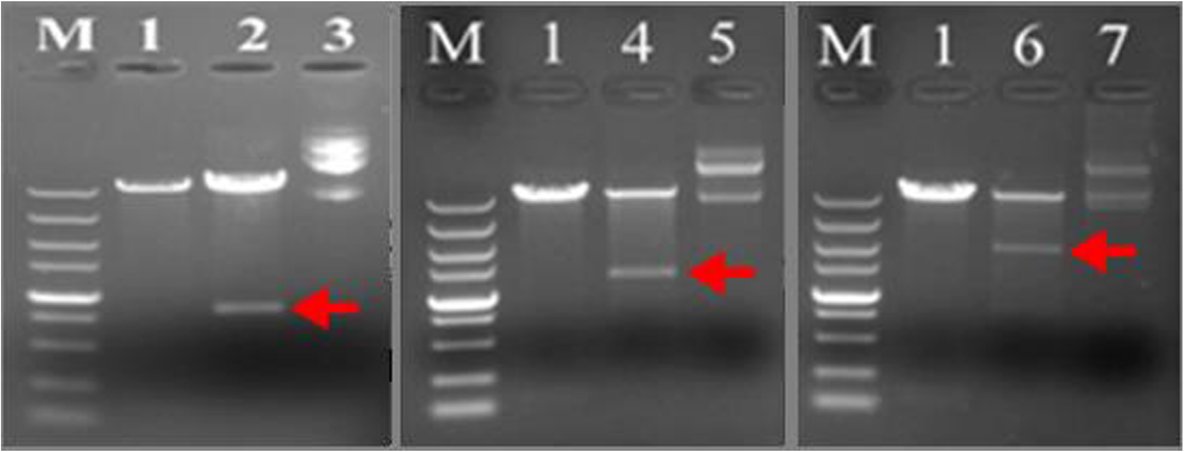
Restriction endonuclease analysis of expression vectors of hELPs-RADA-16. M: DL5000; 1: pET28a (+) digested by *Nco* I& *Xho* I; 2: pET28a (+)-hELP36-RADA-16 digested by *Nco* I& *Xho* I; 3: plasmids DNA of pET28a (+)-hELP36-RADA-16; 4: pET28a (+)-hELP60-RADA-16 digested by *Nco* I& *Xho* I; 5: plasmids DNA of pET28a (+)-hELP60-RADA-16; 6: pET28a (+)-hELP96-RADA-16 digested by *Nco* I& *Xho* I; 7: plasmids DNA of pET28a (+)-hELP96- RADA-16

The coding sequences of hELP36-RADA-16, hELP60-RADA-16 and hELP96- RADA-16 were cut from the corresponding clone vector with *Nco* I and *Xho* I, recovered and recombined with pET28a (+) linearized by same enzymes. Subsequent digestion of *Nco* I and *Xho* I showed a product marked by red arrows between 1000 bp and 750 bp for 36R, about 1200 bp for 60R and about 2000 bp for 96R respectively, suggesting that sequences coding for hELP36-RADA-16, hELP60-RADA-16 and hELP96-RADA-16 was successfully subcloned into the expression vector pET28a(+). Three vectors pET28a(+)-hELP36-RADA-16, pET28a(+)-hELP60-RADA-16 and pET28a(+)-hELP96-RADA-16 for the expression of 36R, 60R and 96R were successfully constructed (Fig. 2).

### Expression and qualitative analysis of recombinant hELPs-RADA-16

**Fig. 3:**
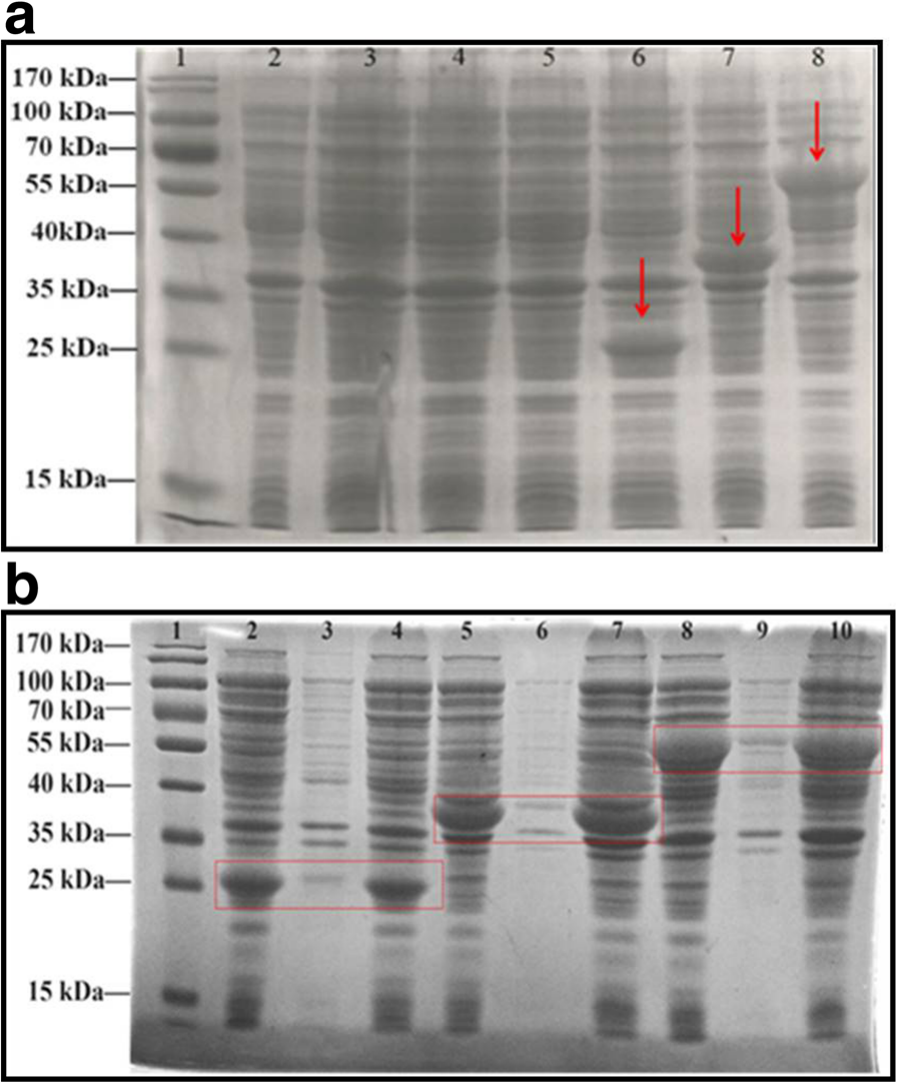
Expression and solubility analysis of hELPs-RADA-16 expressed in BL21 (DE3) by SDS-PAGE. **a** Expression of hELPs-RADA-16 expressed in BL21 (DE3). Lane 1: Protein marker; Lane 2: IPTG-induced cells transformed by pET28a (+); Lane3–5: cell lysis of uninduced cells transformed by expression vectors of 36R, 60R and 96R, respectively; Lane 6–8: cell lysis of IPTG-induced cells transformed by expression vectors of 36R, 60R and 96R, respectively. **b** solubility analysis of hELPs-RADA-16. Lane1: Protein marker; Lane 2–4: supernatant, precipitate and total proteins of ultrasonic cracking cell lysis of BL21 (DE3) transformed by pET28a (+)-hELP36-RADA16; Lane5–7: supernatant, precipitate and total proteins of ultrasonic cracking cell lysis of BL21 (DE3) transformed by pET28a (+)-hELP60-RADA16; Lane8–10: supernatant, precipitate and total proteins of ultrasonic cracking cell lysis of BL21 (DE3) transformed by pET28a (+)-hELP96-RADA16

**Fig. 4:**
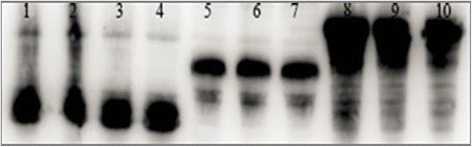
Qualitative analysis of hELPs-RADA16 by Western blotting. Lane1–4: cell lysis of BL21 (DE3) transformed by pET28a (+)-hELP36-RADA-16; Lane 5–7: cell lysis of BL21 (DE3) transformed by pET28a (+)-hELP60-RADA-16; Lane8–10: cell lysis of BL21 (DE3) transformed by pET28a (+)-hELP96-RADA-16

SDS-PAGE detection showed that the recombinant hELPs-RADA-16 including 36R, 60R and 96R could be over-expressed in the host cells of *E. coli* BL21 (DE3) under IPTG induction (Fig. 3a, bands 6–8 indicated by red arrow) compared to the corresponding uninduced samples (Fig. 3a, the bands 3–5) and those prepared from the induced cell culture transformed by pET28a (+) (Fig. 3a, the band 1). The molecular weight (MW) of the specific band with a high signal from Coomassie brilliant blue was approximately 26 kDa, 36 kDa and 60 kDa and consistent with the theoretical MW value of 36R, 60R and 96R, respectively. Solubility analysis showed that 36R, 60R and 96R were expressed and accumulated as soluble rather than inclusion bodies in BL21 (DE3) cells (Fig. 3b, indicated by red box). Western blotting analysis demonstrated that we obtained fusion proteins (Fig. 4).

### Purification and preparation of recombinant proteins

**Fig. 5:**
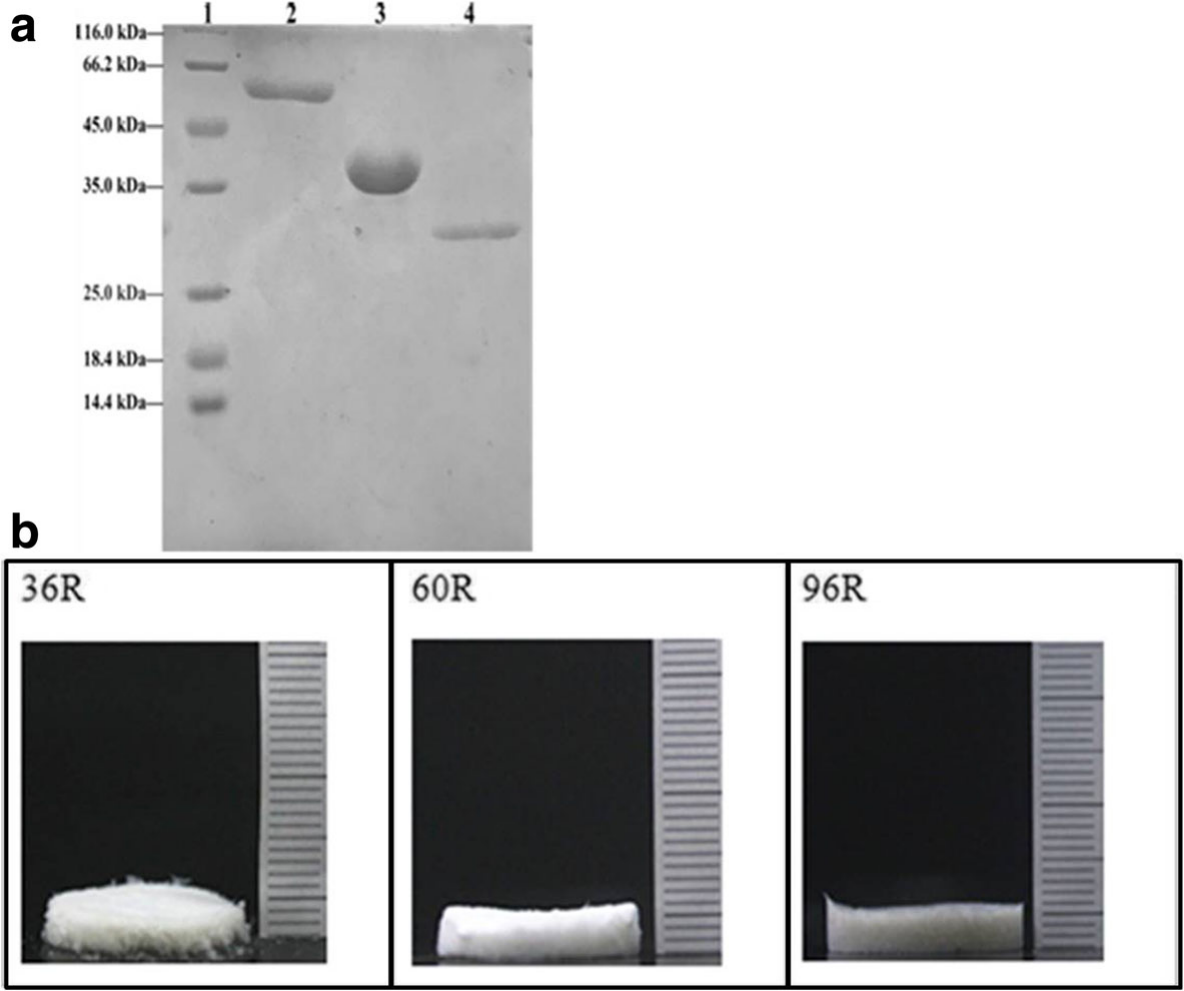
Purified products of recombinant proteins visualized by SDS-PAGE and morphological observation of the products. **a** SDS-PAGE showed purified products of recombinant proteins. Lane 1: protein marker; Lane 2: 96R; Lane 3: 60R; Lane 4: 36R. **b** morphological observation

For the purification of fusion proteins designed in this work, ITC and His-tag mediated affinity chromatography were combined to isolate target proteins from the cell lysis. The purity of prepared 36R was 94.72% and the yield coefficient was approximately 43.5%, the purity of 60R was 96.91% and the yield coefficient was approximately 56.7%. For 96R, the purity was 96.37% and the yield was 47.02%. The purified proteins were visualized by SDS-PAGE (Fig. 5a).

The spongy film of lyophilized 36R, 60R and 96R was white, with approximately 3.2 mm thickness (Fig. 5b). Morphological observation showed that the spongy film of 96R was relatively compact, while the film was unconsolidated for 60R and especially for 36R, from which the mechanical strength of 96R was a bit higher than that of 60R or 36R.

### Cell toxicity test

**Fig. 6:**
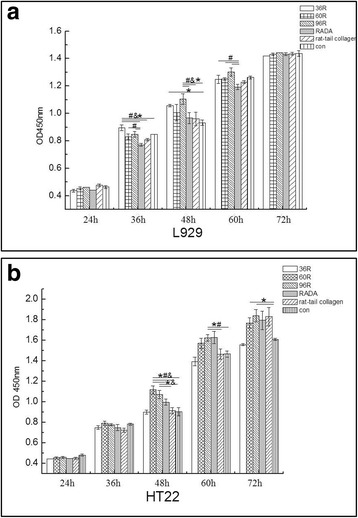
Effects of hELPs-RADA-16 on the proliferation of L929 (**a**) and HT22 cells (**b**). * *P* < 0.05, compared to negative control, # *P*<0.05, compared to RADA-16, *N* = 6; & *P*<0.05, compared to rat-tail collagen; analyzed by one way ANOVA with post hoc Scheffe test, *N* = 6

To investigate the cell toxicity of the recombinant proteins, the mouse fibroblast cell line L929 and hippocampus neuron cell line HT22 were inoculated into wells coated with 1.5 μg/mL 36R, 60R and 96R solution respectively. For all treatments, HT22 and L929 cells adhered to the well bottom after 12 h culture and stretched to display spindle shape. CCK-8 assay of L929 cells showed no significant difference between the wells coated with 36R, RADA-16 and rat-tail collagen after 24 h, and 72 h culture, although significant differences were observed between the treatment group, negative control and positive controls when culture time reached 36 h–60 h (Fig. 6a). Similar results were obtained from HT22 cell line (Fig. 6b). These results indicated that the recombinant proteins 36R, 60R and 96R did not exhibit toxicity effects on the proliferation of L929 and HT22 cells.

### Hemostasis effects of recombinant proteins

**Fig. 7:**
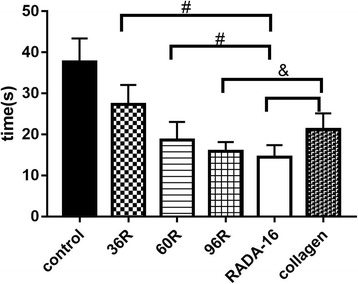
Hemostasis time. ## *P*< 0.01, #*P*<0.05, compared to RADA-16; &, *P*<0.05, compared to rat-tail collagen

**Fig. 8:**
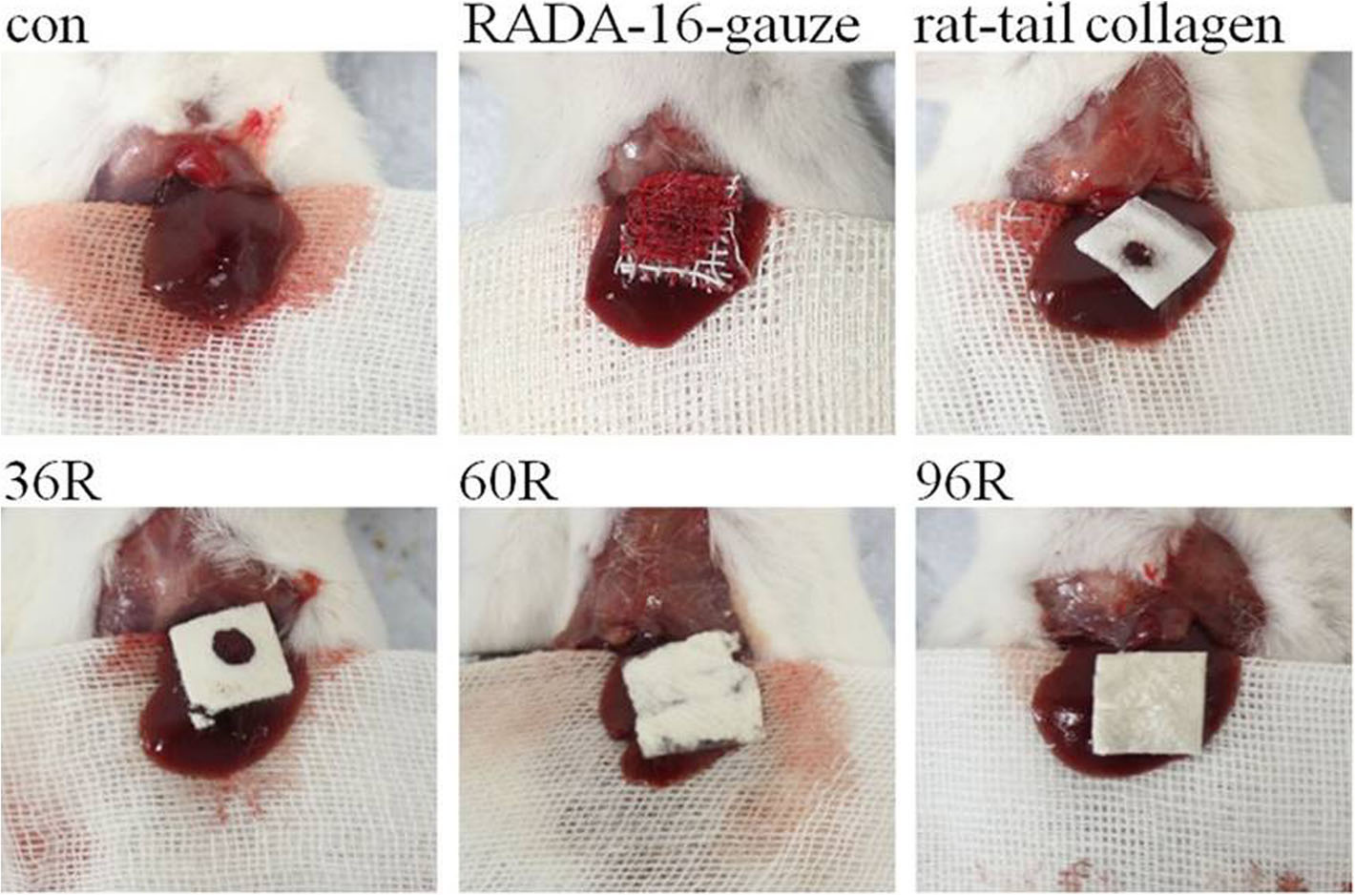
Morphological observation of the hemostatic effect of protein films. Con: negative control; RADA-16: lyophilized gauze immersed with RADA-16

The spongy film of 36R, 60R, 96R, rat-tail collagen and the lyophilized gauze immersed with RADA-16, respectively, was used to stop the wound bleeding of mouse liver, and the hemostasis time was measured. The hemostasis time was 27.21 ± 1.84 s, 18.65 ± 1.97 s, 15.85 ± 1.21 s, 21.23 ± 1.84 s and 14.44 ± 1.33 s for 36R, 60R, 96R, rat-tail collagen and lyophilized gauze immersed with RADA-16, respectively. Visually, the hemostasis time for 36R, 60R or 96R was longer than the lyophilized gauze immersed with RADA-16, and among the three recombinant proteins, the best was 96R. Statistical analysis showed that significant differences were not observed between 96R and the lyophilized gauze immersed with RADA16. However, the difference between 36R/60R and the lyophilized gauze immersed with RADA-16 was significant (Fig. 7). Compared to the spongy film of rat-tail collagen, the hemostatic time of 36R was longer, and the time for 60R or 96R was less than rat-tail collagen. Statistical analysis revealed a significant difference between 96R and rat-tail collagen. Morphological observations showed that the hemostatic effect of 96R was not similar to collagen or lyophilized gauze immersed with RADA-16 when it was attached to the bleeding wound. Blood easily permeated the lyophilized gauze immersed with RADA16 and the spongy film of rat-tail collagen (Fig. 8).

## Discussion

In this work, ITC and His-tag affinity chromatography were combined to isolate 36R, 60R and 96R from cell lysis of transgenic *E.coli* BL21 (DE3). For many researchers, ITC is the most attractive purification approach for ELPs [27]. Using this method, ELPs dissolves in solution when the temperature is lower than its Tt; when the temperature is higher than its Tt, ELPs will aggregate and precipitate, which can be separated from the solution by centrifugation. The ELPs precipitate can re-dissolve by resuspending, but some heat-labile proteins cannot re-dissolve at a temperature lower than Tt. The resuspended solution of ELPs is treated at Tt, followed by centrifugation to give relatively pure ELPs. However, ELPs can re-dissolve very quickly once the temperature is lower than the Tt, due to the transition of ELPs from soluble to insoluble form over a very narrow temperature range [28], from which greater loss of target proteins may occur. In addition, the aggregation of ELPs at temperature ≧ Tt depends on the hydrophobic interaction of peptides, as the sequence of ELPs is composed of the hydrophobic amino acid Val and the neutral amino acids Gly, Ala and Pro. When the temperature is higher above Tt, some contaminant peptides can co-aggregate with ELPs through protein-protein interaction. To obtain ELPs with high purity from a fermented culture for biomedical application, it is essential to develop an effective purification method for ELPs. Consummation of ITC not only depends on high concentration of ELPs, high concentration of salt or long peptides but also depends on the hydrophobic interaction of peptides, which will result in co-aggregation of contaminant peptides. To obtain ELPs with high purity from cell lysis of BL21 (DE3), we employed ITC to remove unwanted proteins, and the coarse pure proteins were further purified using His-tag affinity chromatography. The results showed that the purification method for ELPs used in our work was very effective, which might provide a valuable reference for the purification of other ELPs or ELPs fusion proteins. However, this result also revealed that the hELPs portion in fusion protein possessed phase transition property, but the Tt was high which decreased the efficiency of ITC cycles. We assumed this deficiency could be improved by modifying the amino acid composition and the peptide length of hELPs in future work.

For biomaterials, biocompatibility is essential and highly regarded. The biocompatibility, functions of ELPs and RADA-16 have been investigated and demonstrated intensively [13, 29–31]. However, the alteration of the amino sequence, even by only one amino acid, may profoundly affect the physico-chemical properties of peptides, e.g., normal hemoglobin and that of Sickle thalassaemia patients [32]. To evaluate the effect of fusion manipulation on the biocompatibility of 36R, 60R and 96R, cytotoxicity experiments were conducted on cell level in this work.

Depending on the self-assembly, RADA-16 aqueous solution has been successfully developed to create a novel hemostatic commodity (pre-filled syringe) for clinical application. Once the RADA-16 aqueous solution was added to the bleeding wound, the peptides quickly self-assemble to form a layer of nanometer fiber, which acts as a barrier to effectively block hemorrhages in less than 15 s [10, 11]. This property was expected for 36R, 60R and 96R at the start of work. We attempted to use the aqueous solution to perform hemostatic experiments. However, the 36R, 60R or 96R aqueous solution did not self-assemble like RADA-16 to form hydrogels. To evaluate the hemostatic effect, the recombinant proteins were lyophilized in the wells of poly-tetrafluoroethylene dishes to form a spongy film with certain thickness. The gauze submerged with RADA-16 solution was lyophilized at the same time because RADA-16 would form disintegrating slag after lyophilization. Compared with the commercial product of RADA-16, 36R, 60R or 96R could not self-assemble into nanofilms as RADA-16 does, which were beyond our original expectations, although the fusion protein 96R was better than rat-tail collagen refer to hemostatic effect. This might result from the low proportion of RADA-16 in fusion protein or hydrophobicity exhibited by the ELP peptide in the fusion protein.

## Conclusion

In this work, new biomolecules with hemostatic effects, 36R, 60R and 96R, were created by fusing the gene coding for RADA-16 to the 3′-end of the ELP-encoding ORF. The fusion proteins with purity ≧94% could be produced and purified via cell lysis of a transgenic bacteria culture. The spongy film of the purified 96R exhibited an exciting hemostatic effect better than rat-tail collagen and approaching that of RADA-16.

## Additional files

Additional file 1: The sequencing result of the coding region of RADA-16 in the C-terminus of 36R. This result indicated the coding sequence of RADA-16 in cloning vector pMD19-T-hELP36-RADA-16. (AB1 291 kb)

Additional file 2: The sequencing result of the coding region of RADA-16 in the C-terminus of 60R. This result indicated the coding sequence of RADA-16 in cloning vector pMD19-T-hELP60-RADA-16. (AB1 292 kb)

Additional file 3: The sequencing result of the coding region of RADA-16 in the C-terminus of 96R. This result indicated the coding sequence of RADA-16 in cloning vector pMD19-T-hELP96-RADA-16. (AB1 292 kb)

## Abbreviations

BCA: Bicinchoninic acid
*E.coli*: *Escherichia coli*
ELPs: Elastin-like polypeptides
hELPs- RADA-16: human elastin-like polypeptides fusion RADA-16
IPTG: Isopropylthio-β-D-galactoside
ITC: Inverse phase transition cycles
LB: Lysogeny broth
MW: Molecular weight
PVDF: Polyvinylidene fluoride membrane
SDS-PAGE: Sodium dodecyl sulfate polyacrylamide gel electrophoresis
Tt: Transition temperature
